# Supplementary material for: Mapping the evolution of fertility support policies in China: A content and instrumental analysis
Source: PLoS One. 2025 Oct 9;20(10):e0332137. doi: 10.1371/journal.pone.0332137 (PMC12510515; doi:10.1371/journal.pone.0332137)
Supplement: S1 Appendix — (ZIP) [file pone.0332137.s001.zip › S1 Appendix. 226 original policy documents/14-国家人口和计划生育委员会关于印发人口和计划生育事业发展“十二五”规划的通知(FBM-CLI-4-172616).doc]

国家人口和计划生育委员会关于印发人口和计划生育事业发展“十二五”规划的通知

**发布部门：** [国家人口和计划生育委员会(含国家计划生育委员会)(已撤销)](https://www.pkulaw.com/law/chl?Aggs.IssueDepartment=60304) [机构沿革](javascript:void(0))

**发文字号：**人口发规〔2012〕19号

**发布日期：**2012.03.16

**实施日期：**2012.03.16

**时效性：** [现行有效](https://www.pkulaw.com/law/chl?Aggs.TimelinessDic=01)

**效力级别：** [部门工作文件](https://www.pkulaw.com/law/chl?Aggs.EffectivenessDic=XE0303)

**法规类别：** [人口计划管理](https://www.pkulaw.com/law/chl?Aggs.Category=09701) [计划生育管理](https://www.pkulaw.com/law/chl?Aggs.Category=09705)

国家人口和计划生育委员会关于印发人口和计划生育事业发展“十二五”规划的通知
（人口发规〔2012〕19号）

各省、自治区、直辖市人口计生委，计划单列市、新疆生产建设兵团人口计生委，解放军、武警部队计划生育领导小组办公室，中直机关、中央国家机关人口计生委，委机关各单位、各直属联系单位：
　　现将《人口和计划生育事业发展“十二五”规划》印发你们，请结合实际，认真贯彻落实，确保“十二五”时期人口和计划生育事业发展目标和任务的完成。

国家人口和计划生育委员会
二○一二年三月十六日

　　附件：

人口和计划生育事业发展“十二五”规划

　　本规划根据《[中华人民共和国国民经济和社会发展第十二个五年规划纲要](https://www.pkulaw.com/chl/603c7a7f2158b52bbdfb.html)》、《中共中央[国务院关于全面加强人口和计划生育工作统筹解决人口问题的决定](https://www.pkulaw.com/chl/5e7b01010a4102ffbdfb.html)》（中发〔2006〕22号）和《国家人口发展“十二五”规划》（国发〔2011〕39号）编制，主要阐明“十二五”时期人口和计划生育事业发展思路、目标和主要任务，是全面做好人口和计划生育工作的重要依据。

　第一章　发展环境

　第一节　“十一五”主要成就

生育水平保持稳定。“十一五”时期人口生育水平和变动态势基本平稳，全国总和生育率稳定在1.8以下。人口出生政策符合率在83%以上，人口自然增长率控制在6‰以内，2010年末全国总人口为13.4亿人，总人口控制在13.6亿人以内的规划目标顺利实现。
　　优生优育取得重要进展。启动优生促进工程，开展婴幼儿早期发展服务、独生子女社会行为教育试点。开展宣传倡导、健康促进、优生咨询、高危人群指导、孕前优生健康检查、均衡营养等出生缺陷一级预防工作，并在18个省的100个县启动国家免费孕前优生健康检查项目试点，推进出生缺陷预防关口前移。
　　出生人口性别比攀升势头得到初步遏制。深入开展关爱女孩行动，强化社会性别平等宣传倡导。推广住院分娩实名登记制度，加强孕产期全程服务管理和出生性别统计监测。开展出生人口性别比偏高问题专项治理活动，强化重点省、重点县管理，推动区域协作，查处非医学需要的胎儿性别鉴定和选择性别人工终止妊娠（以下简称“两非”）的行为。2009年全国出生人口性别比升高势头开始得到初步遏制，2010年下降到117.9。
　　流动人口计划生育服务管理迈出重要步伐。国务院颁布实施了《[流动人口计划生育工作条例](https://www.pkulaw.com/chl/f6b2c35d55b7a40ebdfb.html)》。各级人口计生部门流动人口服务管理机构得到加强，推进泛长三角、泛珠三角、环渤海、西北四大区域以及人口流出与流入集中地区之间的区域协作，全国流动人口计划生育服务管理“一盘棋”机制基本建立。深入开展流动人口关怀关爱活动，流动人口免费计划生育服务覆盖面不断扩大，全员流动人口统计信息工作和动态监测调查启动实施。
　　人口计生利益导向政策体系不断完善。农村部分计划生育家庭奖励扶助制度、西部地区“少生快富”工程和计划生育家庭特别扶助制度全面实施，提高了奖励扶助标准，扩大了对象范围。社会经济政策与人口计生政策的衔接得到加强，计划生育法定奖励优待政策得到进一步落实。计划生育保险试点取得初步成效。部分省（区、市）出台了城镇独生子女父母老年奖励政策。“生育关怀行动”普遍开展，“幸福工程”深入推进。
　　宣传教育能力明显提高。更新宣传教育理念，改进宣传教育方式，宣传教育项目执行、活动组织、传播、宣传品开发、培训交流能力提高。婚育新风进万家活动、新农村新家庭计划、新家庭文化屋建设等品牌项目不断发展，依托“7.11”世界人口日等开展大型人口计生社会宣传活动。人口文化建设扎实推进，农村人口文化大院蓬勃发展，少数民族语言宣传品开发取得进展。党政干部人口理论教育积极推进。
　　优质服务能力显著增强。“十一五”农村计划生育服务体系建设任务如期完成，县乡服务站标准化、规范化建设成效显著。实施人口计生科技“三千人才工程”，深入开展优质服务先进单位创建和科技大练兵等活动，计划生育优质服务提质提速，技术队伍结构和素质得到改善。避孕药具不良反应监测网络建成运行。计划生育药械市场得到清理和整顿。地震灾区再生育技术服务成就显著。
　　队伍职业化建设全面推进。开展人口计生工作者职业道德教育。制定人口计生队伍职业标准，建立相关职业资格认证制度和任职资格评定体系。开展生殖健康咨询师培训和考试工作，人口协管员职业申报工作进展顺利。探索建立人口计生系统教育培训长效机制，开发系列培训教材，对市县级人员开展大规模教育培训，组织西部乡镇专业技术人员进修实习。在职学历教育取得积极进展。
　　财政投入稳定增长。各级财政对人口计生的投入稳定增长，财政保障能力和水平明显提高，财政为主的人口计生经费投入保障机制基本确立，“人口和计划生育财政投入增长幅度高于经常性财政收入增长幅度”的要求得到落实，实现2010年末各级财政投入人口计生事业费达到人均30元的目标，切实保障了人口计生事业稳定健康发展。
　　依法行政与行风建设成就显著。全面推进依法行政，加强配套制度建设，进一步完善与《[中华人民共和国人口与计划生育法](https://www.pkulaw.com/chl/252f7ce51132230bbdfb.html)》相配套的行政法规和地方法规，“五五”普法规划得到贯彻实施，深入开展便民维权和基层文明执法专项活动，人口计生行政许可行为得到进一步规范。“阳光计生”行动全面开展，开通“12356”计生服务热线，畅通群众沟通渠道，深入推进基层人口计生政务和村务公开。全面加强廉政风险管理，行风建设和反腐倡廉工作取得新的成就。
　　人口计生综合改革扎实推进。出台了深化人口计生综合改革创新体制机制的指导意见。优质服务先进单位评估改革顺利完成，人口计生目标考核改革继续深化，县乡服务机构管理机制改革稳妥推进。深化行政审批制度改革，行政执法有关制度和程序逐步建立和完善。全面推进诚信计生和基层计生群众自治。部分地方人口计生条例修订完善。
　　决策与科技支撑能力进一步提升。人口发展功能区等重大研究取得重要成果。人口宏观管理与决策信息系统一期工程竣工验收，为全国人口计生信息系统的统一建设和应用奠定了基础。实施全员人口信息管理，初步实现省级数据集中，全国已集中人口个案超过12.5亿。人口计生领域有关的国家973计划、国家重大科技支撑计划、科技基础条件平台建设、人类遗传资源库建设取得重要进展。
　　国际交流与合作巩固发展。积极参与国际人口和发展领域国际事务的讨论，双边、多边合作机制得到巩固和加强。广泛开展国际合作项目，充分发挥前瞻性、探索性和示范性作用，服务国内人口计生中心工作。对外宣传为人口计生工作创造了良好的国际环境。在人口领域南南合作中的骨干和引领作用不断加大，与多个发展中国家签署并成功实施合作备忘录，我国在发展中国家人口领域的影响力、亲和力增强。
　　专栏一 “十一五”主要量化指标进展情况

| 指 标 | 2005年 | 2006年 | 2007年 | 2008年 | 2009年 | 2010年 |
| --- | --- | --- | --- | --- | --- | --- |
| 总人口（万人） | 130756 | 131448 | 132129 | 132802 | 133450 | 134091 |
| 自然增长率（‰） | 5.9 | 5.3 | 5.2 | 5.1 | 4.9 | 4.8 |
| 出生人口性别比 | 120.5 | 119.3 | 120.2 | 120.6 | 119.5 | 117.9 |
| 人均事业经费（元） | 18.3 | 18.6 | 23.0 | 27.4 | 33.2 | 38.9 |

　第二节　面临的挑战

稳定低生育水平的工作依然繁重。我国人口多、底子薄，发展不平衡，人均资源占有量低、生态环境总体比较脆弱，随着人口继续惯性增长，人口、资源、环境压力日益突出。生育政策与群众生育意愿之间的差距依然存在，多数地方计划生育群众自治仍处于起步阶段。现有利益导向政策覆盖人群范围小、标准低，其他经济社会政策与人口计生政策的衔接有待加强。稳定低生育水平的政策措施和工作方式有待进一步创新。
　　提高出生人口素质的任务艰巨。我国出生缺陷发生率仍处于较高水平，据估计，我国每年约有20～30万肉眼可见的先天畸形儿出生，加上出生后数月或数年才显现出来的缺陷儿，累计约占当年出生人口总数的4～6%。当前，优生优育政策体系尚不健全，部门间仍缺乏有效衔接协调机制。预防出生缺陷有关的基础研究和应用研究尚待突破，快速、简便、低成本的孕前筛查技术尚待进一步开发，技术标准和规范等有待制定，服务能力有待提高。
　　出生人口性别比偏高问题突出。自20世纪80年代中期以来，我国出生人口性别比持续偏高，近两年升高势头有所遏制，但2010年仍高达117.9，远高于103～107的正常范围。出生人口性别比偏高问题治理的法律和政策有待加强，现有政策导向作用有限，综合治理难度大。部门间与区域间协作机制有待健全，社会性别平等的文化建设亟待加强。
　　人口老龄化问题日益突出。60岁及以上老年人口将从“十一五”年均净增600多万增加到800万以上，“十二五”期间总量将突破2亿，老龄社会将是我们未来必须适应的常态社会。特别是随着第一批独生子女父母进入老年，亟需研究制定相应对策措施。
　　人口空间分布不合理。人口分布与经济发展不协调，与资源环境承载能力不适应。东部沿海地区流动人口规模庞大，中西部大部分地区人口总量压力和留守人口问题突出。城镇化滞后于工业化，人口城镇化率偏低。人口流动日趋复杂，户籍地和现居住地计划生育双向管理不到位。
　　传统家庭功能弱化。家庭规模小型化、成员居住分散化的趋势明显，单人、单亲、无子女和空巢家庭比例上升，家庭在婚姻、养育、养老等方面面临诸多新问题，相应的家庭发展政策体系尚未形成，支撑家庭发展的服务体系亟待建立。
　　人口计生服务管理体系亟需完善。农村计划生育服务管理职能需要进一步拓展，大部分城市基层人口计生机构和队伍建设需要加强。人口计生领域领军人才、拔尖人才、高技能人才稀缺，家庭服务的人才队伍亟待培养。基层人口服务管理条块分割问题突出，全员人口信息采集和更新机制亟待创新。人口计生财政投入不足，与新时期新任务不相适应。
　　统筹人口发展的调控体系有待健全。人口政策与经济社会发展政策的衔接需要继续加强，统筹解决人口问题的领导体制、问责制度、决策协调机制需要进一步完善。相关法律法规滞后于统筹解决人口问题的要求。
　　总体上看，“十二五”是我国经济社会发展的重要战略机遇期，也是人口发展重大转折期和统筹解决人口问题体制机制建设的攻坚期。稳定低生育水平、统筹解决人口问题的任务十分艰巨，必须以高度的历史责任感和使命感，努力开创人口计生事业发展新局面。

　第二章　指导思想、基本原则和主要目标

　第一节　指导思想

高举中国特色社会主义伟大旗帜，以邓小平理论和“三个代表”重要思想为指导，贯彻落实科学发展观，坚定不移地走中国特色统筹解决人口问题的道路，按照“全面做好人口工作”的总体要求，以建设人口均衡型社会为主线，坚持计划生育基本国策，逐步完善政策，稳定低生育水平，提高出生人口素质，降低出生人口性别比，积极应对人口老龄化，优化人口分布，促进人口长期均衡发展，促进人口与经济社会、资源环境相协调。

　第二节　基本原则

坚持以人为本。把促进人的全面发展作为人口计生工作的出发点和落脚点，注重保障和改善民生。依法行政，更加注重利益引导、服务关怀和宣传倡导，提高家庭发展能力。
　　坚持统筹协调。坚持党政主导，加强部门分工合作，注重社会组织参与、群众自我管理，综合运用经济、教育、科技、法律、行政等措施，形成工作合力。
　　坚持科学指导。把握好人口发展不同阶段的特点，适时完善促进人口长期均衡发展的政策，根据不同地区人口发展的主要问题和功能定位，科学确定工作重点、政策导向。
　　坚持改革创新。不断推进人口计生工作的理论创新、管理创新、服务创新、科技创新，积极探索与实践统筹解决人口问题的体制机制。

　第三节　主要目标

低生育水平保持稳定，人口年均自然增长率控制在7.2‰以内，全国总人口控制在13.9亿以内。出生人口性别结构得到有效改善，出生人口性别比下降到115以下。群众基本享有优质的计划生育、优生优育、生殖健康服务。免费孕前优生健康检查项目覆盖所有县（市、区），目标人群覆盖率达到80%，出生缺陷发生风险降低。流动人口计划生育服务覆盖率达到85%。基层诚信计生服务管理机制基本形成。计划生育家庭老人养老保障水平有所提高，家庭发展能力得到提升。
　　专栏二　 “十二五”人口计生事业发展主要指标

| 指 标 | 年 份 | |
| --- | --- | --- |
|  | 2010 | 2015 |
| 总人口（亿人） | 13.4 | 13.9  以内 |
| 人口年均自然增长率（‰） | 7.2以内 | |
| 出生人口性别比 | 117.9 | 115  以下 |
| 免费计划生育基本技术服务覆盖率（%）^①^ | 80 | 100 |
| 计划生育优质服务县（市、区）比例（%） | 60 | 80 |
| 国家免费孕前优生健康检查项目县（市、区）覆盖率（%） | 3.5 | 100 |
| 国家免费孕前优生健康检查项目目标人群覆盖率（%）^②^ | 68 | 80 |
| 流动人口计划生育服务覆盖率（%）^③^ | 70 | 85 |
| 农村计划生育家庭奖励扶助政策落实  及时率（%） | 67 | 85 |
| 全员人口信息覆盖率（%） | 93 | 98 |
| 人口计生人才总量（万人）^④^ | 69.5 | 100.8 |

　　注：①计划生育基本技术服务包括避孕节育科普知识宣传和咨询指导，放置取出宫内节育器、皮下埋置，人工流产术、引产术，输卵（精）管结扎术、吻合术，计划生育手术并发症和避孕药具不良反应诊断、治疗，避孕节育随访服务等；
　　②国家免费孕前优生健康检查项目目标人群是指已经实施该项目的地区符合生育政策并计划怀孕夫妇；
　　③流动人口计划生育服务覆盖率是指在现居住地享受免费的计划生育基本技术服务、环孕情检查、计划生育药具等服务的已婚育龄人口占有意愿接受这些服务的流动育龄人口的比例；
　　④人口计生人才是指在人口计生领域从事党政管理、宣传倡导、科学研究、技术服务、信息综合、群众工作、产业发展等七类人才。

　第三章　主要任务

　第一节　坚持计划生育基本国策　稳定低生育水平

　　全面做好人口计生工作。深入贯彻计划生育基本国策，坚持和完善现行生育政策，确保人口中长期战略目标的实现。全面加强基层基础工作，突出做好重点地区和重点人群的人口计生工作。深化人口计生综合改革，着力创新稳定低生育水平、统筹解决人口问题的工作机制和方法，解决影响和制约人口计生工作科学发展的突出问题。
　　加强优质服务。继续推进人口计生公共服务体系标准化、规范化和信息化建设。深入开展计划生育优质服务先进单位创建、示范站创建、科技大练兵等活动，实施“六千人才工程”和“拔尖人才”计划。全面落实服务机构宣传教育、技术服务、药具发放、人员培训、信息咨询、优生指导、随访服务、生殖保健等八项功能，全面开展避孕节育、优生优育、生殖保健相关服务。规范以长效措施为主的避孕方法知情选择，指导群众及时落实避孕节育措施，育龄夫妇享有免费计划生育基本技术服务覆盖率达100%。加强计划生育药具不良反应监测，强化服务机构质量监督管理，规范服务程序和服务行为。深化计划生育药具体制改革，全面落实计划生育免费基本药具制度。
　　加大利益导向。全面落实法律法规规定的计划生育家庭奖励优惠政策。促进经济社会相关政策与人口计生政策的有机衔接，在农业开发、就业、社会保障、扶贫开发、征地补偿、集体收益分配等方面，协调制定对计划生育家庭的倾斜政策。进一步完善以计划生育家庭奖励扶助制度、“少生快富”工程和特别扶助制度为主的优先优惠政策体系，扩大范围、提高奖励扶助标准并建立动态调整机制。充分重视并发挥中国计生协会、中国人口福利基金会等组织在人口计生利益导向机制建设中的重要作用。
　　强化依法行政。认真贯彻落实《[国务院关于加强法治政府建设的意见](https://www.pkulaw.com/chl/e768bb639361a481bdfb.html)》和“六五”普法规划，加强依法行政培训，全面提高行政管理人员的依法行政意识和能力。推进基层人口计生文明执法工程，按照规范化、法制化标准，制定执法基本要求，规范行政执法环节。全面推进行政执法责任制，明确各级人口计生部门及内设机构的行政执法责任与权限，建立健全主体合法、权责明确、程序严密、制度健全、监督有效的执法体制。深入开展依法行政示范乡镇（街道）创建活动。健全监督检查工作机制，定期开展行政执法检查，加强重大案件督办工作，完善自我纠错机制，拓宽社会监督渠道。
　　推进基层群众自治和诚信计生。建立完善人口计生基层群众自治长效机制，充分发挥城乡基层组织和计生协会的作用，依法制定计划生育村民自治章程或村规民约。扎实开展人口计生基层群众自治“万村（居）示范”活动。全面推进诚信计生工作，通过基层组织与群众“双向承诺、互为守信”，提高基层组织计划生育服务管理能力。在城市地区和流动人口集聚地区，积极探索“产业式”、“物业式”、“楼宇式”、“联谊式”等新的计划生育组织形式。

　第二节　加强孕前优生工作　提高出生人口素质

全面实施免费孕前优生健康检查政策。以计划怀孕夫妇为重点，提供健康教育、健康检查、风险评估、咨询指导和追踪随访等免费孕前优生健康检查服务。加大力度，全力扩大项目实施区域，“十二五”期末全国各县（市、区）普遍实施免费孕前优生健康检查政策，城乡符合生育政策并计划怀孕夫妇孕前优生健康检查覆盖率达到80%，有效降低出生缺陷发生风险，提高出生人口素质。
　　提高孕前优生健康检查服务能力。以基层计划生育技术服务人员为重点，加强孕前优生健康检查专项技能培训研修。改善计划生育技术服务机构孕前优生服务用房，提高技术装备水平，着重加强县级服务机构优生检验室建设，配备孕前优生健康检查服务所需设备。建立完善国家和省级计划生育临床检验质量监测指导中心和孕前优生数据中心，指导各级服务机构开展质量控制，提高服务水平。
　　完善出生缺陷预防工作机制。加大宣传倡导力度，推动各级政府更加重视出生缺陷预防工作，更加重视发挥人口计生公共服务网络的优势，开展群体预防。加强部门配合，充分发挥专家作用，动员群众积极参与，积极探索政府主导、部门合作、专家支撑、群众参与的工作机制。

　第三节　降低出生人口性别比　促进社会性别平等

坚持男女平等基本国策。提高社会性别平等意识，推动清理涉及社会性别歧视的政策。指导村（居）民自治组织修订完善自治章程或村规民约，在村集体收益分配方面保障计划生育家庭女儿户的合法权益。在扶贫济困、慈善救助、就学就业、公益养老、贴息贷款、项目扶持等方面对计划生育家庭女儿户予以倾斜。进一步开展“幸福工程”和“春蕾计划”等社会公益性活动，充分发挥非政府组织在贫困母亲救助和女童健康成长帮助中的重要作用。
　　加强出生人口性别比综合治理。建立健全国家和省级部门间协调机制，把促进社会性别平等、综合治理出生人口性别比偏高问题列入地方各级人口计生领导小组成员单位的职责范围。规范人工终止妊娠药品和计划生育手术器械经营管理，完善胎儿性别鉴定和人工终止妊娠登记、孕产期全程服务管理制度。严厉打击“两非”行为。完善出生统计监测体系，以出生医学证明和住院分娩为切入点，全面实施出生实名登记制度，建立部门间出生人口信息收集和共享机制。强化区域协作和重点区域的治理，在出生人口性别比严重偏高的连片地区，建立区域协查制度。在重点区域探索建立跨省区出生人口信息共享制度和出生性别监测预警机制。
　　营造男女平等社会环境。把关爱女孩行动、综合治理出生人口性别比偏高问题作为人口计生宣传教育的重要内容。注意发挥志愿者队伍、城乡社区计划生育专干以及各级计生协会等组织的作用，动员大众媒体和社会力量，大力倡导男女平等、关爱女孩的观念。引导对传统婚嫁、祭祀、宗族传承习俗进行合理扬弃。

　第四节　加强流动人口计划生育服务管理　促进人口分布优化

建立健全流动人口计划生育综合治理机制。认真贯彻落实《[流动人口计划生育工作条例](https://www.pkulaw.com/chl/f6b2c35d55b7a40ebdfb.html)》，按照全国“一盘棋”的要求，巩固完善“统筹管理、服务均等、信息共享、区域协作、双向考核”的流动人口计划生育服务管理机制。加强流动人口计划生育统筹协调机制建设，建立联席会议制度，将流动人口计划生育服务管理纳入地方各级人口计生领导小组成员单位的职责范围，推动部门责任的落实和综合治理格局的形成，明确户籍地和现居住地服务管理职责。加强对省际协作的监督考核，建立健全区域协作制度，逐步建立和完善全国统一的流动人口计划生育服务管理规范、服务标准、相关工作程序和协作平台。完善流动人口计划生育服务管理机构和队伍，健全流动人口计划生育联合执法、婚育证明管理、网络化协作、信息共享等服务管理制度。
　　大力推进流动人口计划生育基本公共服务均等化。根据流动人口聚集情况，进一步完善城乡人口计生服务体系。全面推进流动人口“一站式”、“一证式”、“网格化”服务和管理，通过加强流动人口服务车配置、社区流动人口服务中心建设等多种方式，扩大服务半径，提高服务可及性。把流动人口计划生育基本公共服务纳入当地公共财政支出的预算范围，并基本实现均等化。
　　加强流动人口服务管理支撑体系建设。进一步完善流动人口服务管理信息，全面开展省际网络化协作，实现婚育证明、避孕节育情况、“一孩生育服务登记”等网上办理和通报。完善全员流动人口统计信息工作制度，建立全国流动人口生存发展状况和流向、分布的动态监测体系。逐步健全县级以上人口计生部门流动人口计划生育服务管理机构，配备必需的工作人员；强化村（居）特别是流动人口聚集社区的工作网络建设，形成一支高素质的流动人口协管员队伍；合理利用社会力量，充分发挥计生协会、人口福利基金会等组织的作用，引导流动人口自我教育、自我管理、自我监督、自我服务。
　　加强人口分布优化工作。制定、实施与主体功能区相配套的人口政策，促进形成合理的人口分布格局。引导限制开发区域内人口更多地集聚在县城和中心镇，促进禁止开发区域内减少人口总量。深化对人口空间布局的战略研究，做好人口发展规划和主体功能区规划的衔接协调。研究流动人口服务管理体制机制改革、基本公共服务均等化的思路，提出人口管理服务的综合性政策建议。

　第五节　加强计划生育家庭养老工作　积极应对人口老龄化

加强应对人口老龄化问题的战略和政策研究。针对人口老龄化程度的提高以及独生子女老年父母比例的上升，深化前瞻性研究，加强人口规划、政策与其他经济社会规划、政策之间的相互衔接。统筹考虑人口结构与人口数量、素质、分布的关系，加强人口政策与应对老龄化对策的衔接协调，在处理好人口与经济社会、资源环境关系以及实施好国家人口发展战略的前提下，逐步完善政策，多措并举，充分发挥制度优势，走一条中国特色应对人口老龄化的新路子。
　　完善计划生育家庭养老支持政策。完善和落实城镇独生子女父母年老奖励制度。鼓励各地探索计划生育独生子女户、双女户养老和医疗保障优惠政策，减免基本养老和基本医疗保险个人缴费。推动公共养老服务机构优先吸收计划生育奖励扶助对象，社区义工等服务资源优先向计划生育家庭老人提供。积极探索为独生子女父母、无子女和失能老人等特殊老年群体提供必要的养老服务补贴和老年护理补贴。广泛开展计划生育特别扶助对象养老关怀行动。
　　加强计划生育养老服务支持能力建设。鼓励有条件的地区，充分利用人口计生服务管理资源，加强对计划生育家庭养老支持性服务功能的建设。对无子女和独生子女的高龄老人开展养老服务试点，积极探索适合计划生育家庭特点的养老服务模式。针对独生子女、双女户的老年父母，在预防保健、权益保障、精神慰籍和生活照料等方面开展社区服务试点。

　第六节　提高家庭发展能力　促进家庭和谐幸福

推动建立健全家庭发展政策。稳定家庭功能，在优生优育、家庭教育、子女成才、抵御风险、生殖健康、家庭致富以及养老保障等方面，加快建立和完善提高家庭发展能力的政策体系。加大特殊困难家庭的扶持力度，研究出台有利于促进计划生育家庭成员就学、就业、创业、勤劳致富的扶持政策。将计划生育节育手术并发症三级以上人员纳入计划生育家庭特别扶助制度范围。免费为符合条件的育龄妇女提供再生育技术服务。
　　着力提高家庭服务能力。探索建立以家庭为中心的人口计生公共服务体系，开展婚育指导、家庭初级保健、儿童早期发展、家庭教育指导，以及对计划生育家庭、空巢家庭、流动家庭、留守家庭的关怀服务等。组织实施计划生育生殖健康促进工程，全面加强家庭初级保健工作，促进适龄群众人人享有生殖健康服务目标的实现。在全员人口信息系统中，加强家庭信息采集和管理，为家庭发展政策的制定和实施提供依据。
　　大力推进新型家庭人口文化建设。以婚育新风进万家活动、关爱女孩行动、新农村新家庭计划为载体，广泛宣传人口计生政策。开展幸福家庭创建活动。倡导婚姻自由平等、生殖健康、优生优育、社会性别平等的观念，倡导积极健康、负责任的婚育行为，大力弘扬尊老爱幼、邻里互助的社会风尚。加强流动人口聚集区多元文化交流，促进流动人口融入当地社会。加强农村人口文化设施建设，并纳入新农村建设的整体规划。加强少数民族人口文化建设。

　第七节　夯实基层基础工作　提高人口计生服务管理能力

加强城乡人口计生公共服务体系建设。实施“十二五”人口计生服务体系建设规划，通过改善基础设施，配备优生检测设备，加强流动服务，构建支撑平台，满足人们群众日益增长的人口计生公共服务需求。
　　加强社区网络建设。加强社区资源的统筹和共享，按照常住人口开展计划生育服务管理，引进社会工作理念与方法，对社区工作人员或公益岗位人口计生工作人员进行培训，充实城乡社区服务管理力量。加强流动服务车的配置和城乡社区服务网点建设，充分发挥城乡社区人口计生公共服务网络的作用，探索建立社区人口和家庭服务中心。探索社会化的免费药具发放服务模式，在城乡社区服务中心、医疗机构、药店、便利店等普遍设立药具发放点。
　　加强人口信息体系建设。建设全员人口统筹管理信息系统，为社会管理和公共服务搭建基础平台。全面推进全员人口信息管理，科学监测和评估人口发展状况，及时发布人口总量、结构、分布预测预警信息。以加强人口管理创新为目标，创新全员人口信息采集机制。以全员人口信息为切入点，广泛参与社会管理。积极推进跨地区、跨部门的信息共享，科学采集和更新人口信息。加大对人口计生信息化建设的投入，缩小不同地区间信息化发展差距。
　　加强人才队伍建设。建立健全人才培养、引进、使用、交流、评价、激励机制，实施人才发展重点支持政策和重大人才培养工程，造就一支高素质的人口管理和服务人才队伍，2015年人口计生领军人才达1000人，拔尖人才达2000人，高技能人才达75000人。充分利用高校和研究机构的教育资源，推进继续教育，提高队伍学历层次，积极推进学历证书和职业资格证书制度，建立职业标准、教育培训、评估认证和职业拓展体系。建立相关新职业的培训、认证等制度，大力推进人口管理和家庭服务的人才队伍建设。建立职业化培训教育基地，加快基层人口计生工作人员职业化建设步伐。加强社会工作专业人才队伍建设。

　第八节　开展基础与应用研究　提高人口计生科技创新能力

开展重大基础研究。依托国家“973”计划项目，深入研究人类生殖相关机理，为避孕方法取得突破性进展提供理论支撑。组织较大规模流行病学研究，开展常用节育、生育以及不孕不育诊断治疗相关产品和技术的系统评估，研究避孕节育相关疾病和健康问题的谱系分布与动态变化。以孕前预防为重点，组织开展遗传因素、环境因素与我国重大出生缺陷的关系研究，探索出生缺陷发生机理机制和影响因素。加强人口结构、迁移、老龄化等方面重大问题科技应对研究，力争在中老年生殖健康和计划生育相关技术对中老年人口健康的影响与对策研究方面取得突破。
　　加强新技术新产品开发和应用研究。积极开展生殖健康研究，支持计划生育生殖健康新技术新产品产业化和引入推广。组织生育调节系统研究，开发和改进避孕药具和技术，提高其有效性、安全性，促进避孕药具更新换代。以群体预防为目标，组织开展孕前风险评估和实验室筛查诊断技术研究，研发以孕前-围孕保健为重点的出生缺陷预防关键技术。
　　加快科技创新基地建设。在避孕节育、优生优育、生殖健康等领域，继续加强科技创新基地建设，重点支持国家人类遗传资源中心建设，建设2～3个具有国际水平的计划生育生殖健康科研基地，新建9个部委级重点实验室，争取增建1个国家级重点实验室。支持企业和科研机构开展合作研究，探索形成创新研究开发机制。

　第九节　着力改革创新强化　统筹解决人口问题机制建设

完善综合决策与协调机制。推动各级党委政府把统筹解决人口问题、建设人口均衡型社会提上重大决策议事日程，把人口计生工作纳入改善民生的总体部署。建立人口发展政策协调制度，统筹协调公共政策、公共资源，推动部门间、区域间的相关经济社会政策与人口发展政策的有机衔接。探索建立重大建设项目人口评估机制。重大经济社会政策出台前，开展对人口发展影响的综合评估。
　　完善人口宏观监测与调控体系。研究制定人口长期均衡发展的综合指标体系，加快建立人口发展监测评估体系。加强人口计生统计工作，推动建立部门间人口统计信息沟通机制。健全其他国家专项规划与人口发展规划衔接协调机制，完善以人口发展规划和事业发展规划为基础、各专项规划为支撑的人口发展规划体系。加强年度计划与规划之间的衔接，完善年度人口计划指标体系，建立规划实施跟踪机制。
　　建立统筹解决人口问题的目标管理责任考核体系。改革和完善人口目标管理责任制，探索建立以统筹解决人口问题为导向的考核评估指标体系。逐步将人口发展规划重点任务落实、人口发展相关政策协调、人口信息共享等纳入目标管理责任制考核。科学运用考核结果，更加注重奖励性引导。
　　推进基层人口服务管理创新。在国家综合配套改革试验区开展人口服务管理体制改革试点，推动人口服务管理体制创新。建立市级规划指导、县级协调监督、乡级组织落实、社区综合服务的基层人口工作机制。完善人口计生服务管理体系，优化公共资源配置，以全员人口信息为基础，构建人口管理和公共服务平台。全面推行社区人口网格化管理，建立社区人口信息的统一采集和更新机制，完善社区人口监测功能。

　第四章　规划实施的保障措施

　第一节　加强对人口计生工作的组织和领导

构建党委领导、政府负责、社会协同、公众参与的人口计生工作格局，健全领导机制、协调机制、监督机制和问责机制，将统筹解决人口问题的有关任务纳入各相关部门的职责和重大事项督查范围。坚持人口计生工作党政一把手亲自抓、负总责，对地方党政、相关部门、人口计生部门的目标责任落实情况进行分别考核，并实行“一票否决”制度。

　第二节　完善人口计生相关法律法规

针对人口发展的新情况、新问题，研究提出“十二五”和中长期人口发展法律保障体系建设总体框架。推动修订《[中华人民共和国人口与计划生育法](https://www.pkulaw.com/chl/252f7ce51132230bbdfb.html)》以及[社会抚养费征收管理办法](https://www.pkulaw.com/chl/f03446d289d2b59ebdfb.html)，推动制定促进出生人口性别平衡等方面的法规。各地根据人口发展实际情况，依法、适时、稳妥地修订人口与计划生育条例以及社会抚养费征收、流动人口计划生育管理等方面的规章制度。加大协调力度，促进有关方面在制订和修订相关法律法规时，充分考虑人口发展与计划生育工作的需求。

　第三节　健全人口计生财政投入保障机制

把人口计生列入基本公共服务的重要领域，将计划生育家庭优先优惠政策纳入政府改善民生的总体部署。建立健全“财政为主、稳定增长、分类保障、分级负担、城乡统筹”的人口计生财政投入保障机制，落实财政对人口计生事业投入增长幅度高于经常性财政收入增长幅度的政策；区别不同重大项目分别确定中央和地方资金的分担办法；进一步完善中央财政对地方的转移支付，建立以常住人口为依据进行中央财政对地方的转移支付机制，不断提高中央和省级财政投入比例。

　第四节　加大宣传与国际合作交流力度

深入开展人口国情、人口政策和人口发展规划的宣传，充分发挥新闻媒体作用，正确引导舆论，在全社会营造有利于人口计生工作的氛围。积极参与人口与发展领域国际事务的讨论和规则制定。继续推进人口领域南南合作，探索南北南合作。在应对人口数量、素质、结构、迁徙等挑战方面寻求实质性合作。通过引进理念、借鉴模式，积极推进人口领域的体制机制创新。开展对外宣传和国际倡导，加强系统内国际交流合作能力建设，促进相关科研院所、非政府组织、企事业单位的国际交流与合作。

　第五节　加强人口计生系统行风建设

加强队伍职业道德建设，增强以人为本、诚实守信的观念。全面开展廉政风险防控管理，完善廉政勤政各项制度。逐步建立电子监察系统，重点加强对人口计生行政审批权、执法权和重大项目资金运行的实时监控和检查。深入推进“阳光计生行动”，全面规范政务公开、事务公开、村务公开，扎实开展“请农民兄弟姐妹评计生”、“请流动人口评计生”、“系统内下评上”活动，充分发挥“12356”阳光计生服务热线、行风监督员、实名举报奖励制度和新闻媒体监督的作用。加强重大事项巡视和监督检查工作。

　第六节　加强规划实施的监测评估

各地要根据《国家人口发展“十二五”规划》和本规划，制定本地区人口计生事业发展规划及实施方案，并以此为依据，制订目标管理责任书和年度计划。建立规划实施的监测评估机制和民主决策、公众参与机制。强化对规划执行情况的督导，加大区域性指标的监测和评估力度。加强对重大政策、重大工程项目的动态跟踪和实施效果的评估。完善规划期中、期末评估机制。
　　专栏三　重大基础设施投资工程

全员人口统筹管理信息系统建设工程：建设自主采集、办事采集和部门交换的“三个采集渠道”，完善全员人口信息、业务信息和决策信息“三个信息库”，完善人口计生服务管理、人口统筹管理信息采集和人口决策支持“三个业务系统”，构建规范统一、信息共享、两级处理、六级应用的人口信息服务管理平台，全面支撑实有人口动态管理、跨区域交换、监测分析、行政执法、优生优育、利益导向、事业统计和科学决策等各项业务；充分利用政府已有网络资源，完善网络基础设施；建立人口信息化标准规范；按内外网规划要求，分网部署，分域控制，确保网络安全。

人口计生服务体系建设工程：对未达标的县级服务站（含所在区未设站的市级站）和乡镇中心服务站继续推进标准化、规范化建设，为县级服务站配备孕前优生健康检查设备，继续实施计划生育流动服务车项目，建成11个部委级研发和指导中心。

国家人类遗传资源中心建设工程：组建鉴定、保藏、共享、信息管理、科学研究和工程技术开发等6个专业中心，建设遗传资源筛检鉴定、保藏、科学研究、数据共享与信息挖掘、开放共享、工程技术开发和公共服务等7个功能区，收集和保藏11个重大人类遗传资源库。

重点地区流动人口动态监测与决策支持工程：设立流动人口动态监测与调控关键技术攻关项目，制定引导人口有序迁移、合理分布指标评价体系，构建分地区人口密度动态监测、全员流动人口数据库、人口流动态势模拟与决策支持模型库为一体的人口数据分析平台，推进流动人口动态监测技术集成和决策支持系统示范应用。在流入人口较集中的城市和流出人口较多县设立固定监测点。监测流动人口基础信息和重点信息，建立重点地区流动人口监测机制和预警反应机制。

人口计生科技创新基地建设：依托国家人口计生委科学技术研究所、8个省级科研院所等机构，建设男性生育力监评和保护技术、生育调控技术、非人灵长类生育调节技术评价、出生缺陷预防、优生技术、生殖与遗传医学、男性生殖健康、老年生殖健康重点实验室，增强人口计生领域科技创新能力。

避孕药具不良反应监测与防治科技体系建设：完善监测能力，形成包括全国避孕药具不良反应监测中心和105个监测点构成的不良反应监测网络。建设面向避孕安全的数字化、智能化、网络化、个性化的避孕药具不良反应监测与防治的科技服务网络体系。

人口计生强基提质工程：建设人口计生职业教育学院，开发网络教育平台。通过广播电视和远程教育学院，支持和开展人口计生系统的继续教育。进一步加强与高等院校的合作，鼓励支持设立与统筹解决人口问题相关的研究中心。以国家级培训机构为龙头，以省级培训中心为支撑，充分利用党校、行政学院、高等学校、科研院所的资源，完善人口计生教育培训体系。在部分省（区、市）设立人口计生公共服务体系转型拓展实践考察基地和实习培训基地。

人口和家庭公共服务示范中心建设工程：加强与有关部委和单位沟通协调和合作，建立人口计生老龄服务示范中心、生殖健康咨询研究服务示范中心、儿童早期发展研究服务示范中心，培养、吸引和集聚相关急需紧缺人才；通过鼓励各地试点，上下联动、推动整体发展，探索依托人口计生公共服务网络，开展家庭服务的新模式。

　　专栏四　主要惠民政策

计划生育家庭奖励扶助制度：提高农村计划生育家庭奖励扶助标准并建立动态调整机制，将符合条件的成建制转为城镇户口但未享受城镇居民社会保障和福利待遇的原农村居民、夫妻一方是农村居民、未育收养的夫妻纳入奖励扶助范围。

“少生快富”工程：提高西部地区可以生育三个孩子，只生育了两个或一个孩子的农牧民家庭奖励标准，同时加大在生产、生活方面的政策扶持力度。

计划生育家庭特别扶助制度：提高独生子女死亡伤残家庭特别扶助金标准并建立动态调整机制，将三级以上计划生育节育手术并发症人员纳入计划生育家庭特别扶助制度范围。

免费再生育技术服务制度：建立再生育专项资金，为符合条件的子女死亡伤残且有生育意愿和能力的计划生育家庭，免费提供规定的再生育技术服务。

流动人口计划生育基本公共服务均等化促进工程：为流动人口提供免费计划生育基本技术服务，并为流动育龄妇女免费提供一年一次生殖健康体检，每年按流入地经费支出一定比例对流入地进行补贴。在流动人口集中的社区增设流动人口计生协管员公益岗位，逐步建设5000个标准流动人口基层协会活动中心，给予一定补助。

国家免费孕前优生健康检查项目：在全国建立免费孕前优生健康检查制度，为城乡符合生育政策并准备怀孕的夫妇提供免费孕前优生健康检查服务。

婴幼儿早期发展服务项目：对0-3岁独生子女开展早期发展服务，为农村留守儿童提供扶助。

　　专栏五　服务管理能力建设工程

统筹解决人口问题综合改革项目：根据各地人口发展不同情况、面临的重点热点问题，建设不同层级、类型的人口计生综合改革试验区，探索中国特色统筹解决人口问题的工作模式。以重点区域和省级单位为主体，重点培育、确立100个左右处于不同发展阶段的国家综合改革示范点，着力推进统筹解决人口问题机制建设。

诚信计生：把人口计生工作作为城乡基层社区诚信社会建设重要内容，以诚信承诺为基础，加强基层管理，完善群众自治机制，推动以长效机制措施为主的避孕节育方法知情选择。

万村（居）示范：按照“组织建设好、制度规范好、村（居）务公开好、宣传服务好、计生诚信好、文明建设好”的标准，培育、树立一大批人口计生基层群众自治示范村（居）。

出生人口性别比偏高问题综合治理工程（关爱女孩行动）：通过宣传倡导、利益导向、优质服务、查处“两非”、监测评估，以及立法、社会性别平等评估和出生实名登记等综合措施，推动新型生育观念和文化、社会性别平等观念的形成，最大限度地防范“两非”行为发生。在省际推广建立区域协作治理模式。实施分类指导，重新确定重点省（区）和重点市县。在各省（区、市，除西藏、新疆外）建立综合治理出生人口性别比偏高的试点市县治理模式。采取设立“重点治理年”等多种措施，开展专项督查，全面加大重点省（区）和重点市县的综合治理力度。

人口计生文明执法工程：通过依法行政培训、信息化建设以及行政执法设备建设，创新手段，规范执法，进一步提高人口计生系统依法行政能力和水平。

人口计生职业体系建设工程：开展生殖健康咨询师培训和职业资格认证工作。加强社会工作专业人才队伍建设。建立职业标准体系、学科教材体系、教育培训体系、评估认证体系和职业拓展体系。建设一批职业化培训教育基地。

人口计生管理人员能力建设工程：按照《[中华人民共和国公务员法](https://www.pkulaw.com/chl/823ca97b7f55d18cbdfb.html)》的有关要求，建立各级管理人员的能力胜任模型，加强行政管理人员的教育培训，提高依法行政能力、分析研究能力、综合协调能力、优质服务能力、群众工作能力，适应形势发展和事业需要。

人口计生专业技术人员继续教育工程：落实专业技术人员继续教育每年25学分（72学时）的规定。高级专业技术人员将社会科学、人口和生殖健康领域的前沿和发展趋势作为继续教育内容；中级专业技术人员以推广应用新技术作为主要学习内容；初级专业技术人员加强基本理论、基本知识和基本技能的学习教育和在职学历教育。

人口计生强基固本工程：在地方机构改革中，要确保基层人口计生工作机构、编制、人员和待遇的落实。进一步加强基层人口计生队伍的教育培训，提高依法行政和优质服务水平。县级人口计生工作人员具有本科学历的达到80%，乡级人口计生工作人员具有专科学历的达到80%。

计划生育优质服务能力建设项目：加强避孕节育技术创新和推进应用；建立环情孕情监测服务机制，提高综合避孕率和避孕有效率；开展计划生育优质服务先进单位和示范站的创建活动，提高基层计划生育服务管理水平；实施“六千人才”工程、“拔尖人才”计划，开展科技大练兵活动。

婚育新风进万家活动：在农村建设各具特色的人口文化大院，推进新家庭文化屋建设，在城市和社区完善青少年性健康教育基地。充分利用远程教育资源，扩大远程宣传教育范围。创新方式方法，开发人口文化公益广告和动漫画精品，充分利用现代的多媒体、手机和网络资源，传播新型婚育文化。

新农村新家庭计划：在每省（区、市）选择3-4个试点县，全国总共选择100个县，创建具有生育文明新风尚、优生优育新观念、男女平等新面貌、尊老养老新行动、绿色环保新生活的“五新家庭”，国家每年资助试点县建设10个乡、村示范新家庭文化屋。

少数民族人口文化建设项目：加强对少数民族地区宣教设备和宣教资源建设的投入，改善少数民族地区宣教手段；开展少数民族人口文化发展研究，积极探索发展少数民族新型家庭人口文化建设的方法和途径，重点支持少数民族地区民族语言宣传品和人口文化影视开发；充分发挥宗教人士在少数民族人口文化建设中的重要作用，加强少数民族地区人口文化建设。

青少年健康人格教育工程：主要依托人口计生公共服务网络，对10-24岁青少年人群分层次地开展性与生殖健康基本知识的普及教育。依托社区文化阵地、青少年科技教育基地、人口计生宣传服务机构、计生协会工作网络，重点针对小学、中学、高中和大学生及其家长，开设青少年思想品德和素质教育第二课堂。推动城市社区建立规范的“青苹果之家”咨询服务网络，协助各级广播电视台开设讲座或专题节目，开发科普读物和音像产品，借助媒体和远程教育平台，把青少年健康人格教育内容普及到城乡社区。

© 北大法宝：（[www.pkulaw.com](https://www.pkulaw.com/)) 专业提供法律信息、法学知识和法律软件领域各类解决方案。北大法宝为您提供丰富的参考资料，正式引用法规条文时请与标准文本核对。
欢迎查看所有[产品和服务](https://www.pkulaw.com/helps/2.html)。法宝快讯：[如何快速找到您需要的检索结果？](https://www.pkulaw.com/helps/70.html)[法宝](https://www.pkulaw.com/helps/69.html)V6有何新特色？

扫描二维码阅读原文

**原文链接：**<https://www.pkulaw.com/chl/63383c222d6c19e4bdfb.html>
